# High throughput evaluation of gamma-H2AX

**DOI:** 10.1186/1748-717X-4-31

**Published:** 2009-08-24

**Authors:** Dane Avondoglio, Tamalee Scott, Whoon Jong Kil, Mary Sproull, Philip J Tofilon, Kevin Camphausen

**Affiliations:** 1 Radiation Oncology Branch, National Cancer Institute, National Cancer Institute, Bethesda, Maryland USA; 2 Drug Discovery Program, H. Lee Moffitt Cancer Center, Tampa, Florida USA

## Abstract

The DNA double-strand break (DSB) is the primary lethal lesion after therapeutic radiation. Thus, the development of assays to detect and to quantitate these lesions could have broad preclinical and clinical impact. Phosphorylation of histone H2AX to form γ-H2AX is a known marker for irradiation-induced DNA DSBs. However, the first generation assay involves the use of immunofluorescent staining of γ-H2AX foci. This assay is time consuming, operator dependent and is not scalable for high throughput assay development. Thus, we sought to develop a new assay using a high throughput electrochemiluminescent platform from Mesoscale Discovery Systems to quantify γ-H2AX levels. The results show that our assay utilizes significantly less time and labor, has greater intra-assay reproducibility and has a greater dynamic range of γ-H2AX versus irradiation dose.

## Introduction

Because the DSB is the critical lesion induced by ionizing radiation in terms of cell killing, their analysis provides essential insight into fundamental and translational radiobiology. However, DSBs are relatively infrequent as compared to the other radiation-induced lesions such as SSB and base damage, resulting in technical challenges in the development of specific analytical procedures. Standard techniques for quantifying DSB induction and repair have included pulsed field gel electrophoresis (PFGE) and the neutral comet assay [1]. Over the last several years, γ-H2AX expression has been established as a sensitive indicator of DSBs [2]. At sites of radiation-induced DNA DSBs, the histone H2AX becomes rapidly phosphorylated (the phosphorylated form is referred to as γ-H2AX) forming readily visible nuclear foci [2,3]. Although the specific role of γ-H2AX in the repair of DSBs has not been defined, recent reports indicate that the dephosphoryation of γ-H2AX and dispersal of γ-H2AX foci in irradiated cells correlates with the repair of DNA DSBs [4-6]. Moreover, Macphail et al in their study of ten cell lines reported that the loss of γ-H2AX correlates with clonogenic survival after irradiation [7].

Currently, immunofluorescent staining is one method for evaluation of γ-H2AX [8]. However, the assay typically involves the manual counting of nuclear foci, with each focus containing γ-H2AX molecules. The assay also has a limited dose range and is not amenable to high throughput screening (HTS). γ-H2AX may also be evaluated by immunoblot assay but this technique is time and labor intensive, has a fairly narrow range of detection, and also is not scalable to HTS. Lastly, flow cytometry has been used to analyze γ-H2AX; however, flow cytometry methods are not readily integrated into HTS. Although each of the aforementioned methods of evaluating γ-H2AX is effective and has provided important information, there is still a need for an analytical high throughput assay that is capable of screening radiomodifying drugs across diverse cell lines and *in vivo *tissue. We show that using an electrochemiluminescent detection system, γ-H2AX can be evaluated in both cultured cell lines and *in vivo *murine tissue with an efficient, reproducible methodology that is scalable for HTS [9].

## Materials and methods

### Cell lines and treatment

The human glioblastoma cell line (U251) and pancreatic cell line (MiaPaca) were obtained from the National Cancer Institute Frederick Tumor Repository. The breast tumor cell line variant MDA-MB-231BR was supplied by the laboratory of Patricia Steeg (National Cancer Institute, Bethesda, MD). Cells were grown in DMEM (Invitrogen) with glutamate (5 mmol/L) and 10% fetal bovine serum, and maintained at 37°C, 5% CO_2_. 17DMAG and perifosine, provided by the Developmental Therapeutics Program of the National Cancer Institute, were reconstituted in DMSO (100 mmol/L) and PBS (100 mmol/L) respectively, and stored at -20°C. Cells were irradiated using a Pantak X-ray source at a dose rate of 2.28 Gy/min.

### Clonogenic assays

Cultures were trypsinized to generate a single cell suspension and a specified number of cells was seeded into each well of a six-well tissue culture plate. After allowing cells time to attach (4 h), cultures received 17DMAG (50 nmol/L) and perifosine (9 μmol/L) or DMSO (vehicle control) for 16 h before irradiation: media was then removed and replaced with drug-free media. Ten to fourteen days after seeding, colonies were stained with crystal violet, the number of colonies containing at least 50 cells was determined, and surviving fractions were calculated.

### Immunofluorescent staining for γ-H2AX

Immunofluorescent staining and counting of γ-H2AX nuclear foci was performed as previously described [9]. Slides were examined on a Leica DMRXA fluorescent microscope. Images were captured by a Photometrics Sensys CCD camera (Roper Scientific) and imported into IP Labs image analysis software package (Scanalytics, Inc.). For each treatment condition, γ-H2AX foci were determined in at least 50 cells. Cells were classified as positive (i.e., containing radiation-induced γ-H2AX foci) when more than five foci were detected.

### MSD Direct Coat Assay

Cells were grown and treated on 100 mm plates. After specified treatments and incubations, cells were harvested: scraped into PBS, washed, and frozen overnight at -80°C. Each cultured condition was resuspended in lysis buffer containing NaCL (500 mM), EDTA (2 mM), Triton X-100 (1%), sodium deoxycholate (1%), SDS (1%), Tris HCl (50 mM), NaF (10 mM), phosphatase and protease inhibitors (1×), and PMSF (2 mM). Proteins were solubilized by sonication, concentrations determined by Bradford assay, and lysates coated onto MSD high bind plates. Wells were blocked with 3% blocking solution, washed, and a sulfo-ester tag conjugated phospho-H2AX (Abcam) detection antibody was added in 1% blocking solution (1 μg/ml). Wells were washed 3× and 1× MSD Read Buffer was added before analysis in a MSD Sector Imager 2400.

### Animal Methods

All animal studies were conducted in accordance with the principles and procedures outlined in the NIH Guide for the Care and Use of Animals. Four to six week old nude mice were injected subcutaneously with U251 cells (1 × 10^6^) on the lateral aspect of the rear leg. When tumors reached 500 mm^3 ^mice were irradiated. Mice were sacrificed, tumors extracted, tissue homogenized, and resuspended in lysis buffer. The MSD Assay was carried out as stated above.

### Statistical analysis

*In vitro *experiments were repeated thrice and statistical analysis was done using a Student's *t *test. Data are presented as mean ± SD. A probability level of a *P *value of < 0.05 was considered significant.

## Results and Discussion

**Figure 1:**
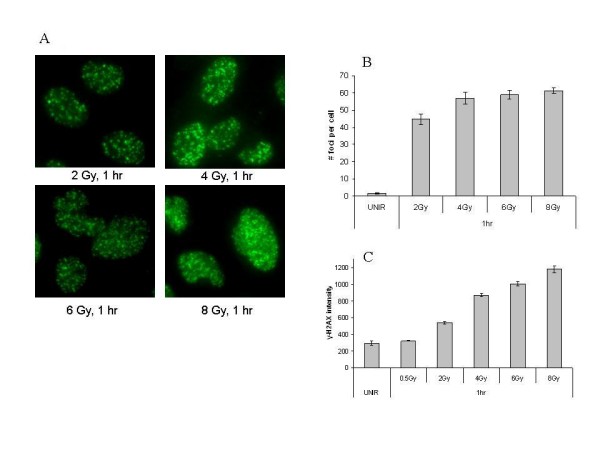
**γ-H2AX evaluation post-irradiation**. (A) U251 cells were plated onto chamber slides, irradiated at the specific doses, and fixed for immunocytochemical analysis. Foci were evaluated three times in 30 nuclei per treatment per experiment. (B) Plot of the linear dynamic range of the immunofluorescent staining assay. (C) Plot of the linear dynamic range of the MSD Assay.

**Figure 2:**
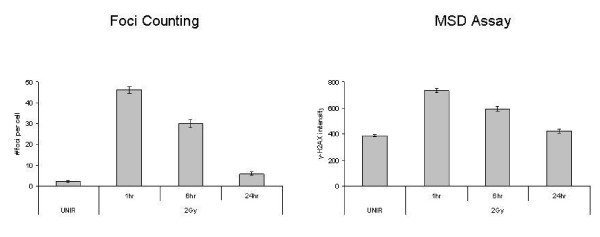
**Comparison of foci versus MSD for the evaluation of DNA-DSB repair**. The U251 cell line was plated out onto chamber slides for the immunofluorescent assay in which foci counting was utilized. U251 cells were plated onto 100 mm plates for the MSD assay. Irradiation was carried out and the respective assays were performed at designated time points.

A critical determinant of radiation-induced cytotoxicity is the induction and repair of DNA damage, specifically DSBs [10]. The two main confounders to the current γ-H2AX foci assay are the manual quantitation and the limited range of the assay. To demonstrate the more limited dose range, immunofluorescent staining was performed on U251 cells 1 hour post irradiation at doses from 0-8 Gy (Figure 1). This figure and all that follow is a representative figure from one of three independent experiments. As shown, exposures higher than 4 Gy result in foci saturation, reducing the useful range of the assay to doses less than 4 Gy. As a measure of linearity, we calculated the R squared value for figure 1B as 0.815. Comparatively, as shown in figure 1C, the γ-H2AX MSD assay (96-well format) has a linear dynamic range up to 8 Gy (R squared value is 0.967) with a high intra-assay reproducibility. Though manual foci counting may be more sensitive than the MSD assay (larger difference in values between unirradiated and 2 Gy), the MSD assay data reflect a greater linear dynamic range. To determine whether the results derived from the MSD system were broadly applicable, additional cell lines (MDA-MB-231BR and MiaPaca) were irradiated and assayed for γ-H2AX at varied doses of IR. Reproducible γ-H2AX levels that were dose dependent were derived for the two additional cell lines (data not shown). In addition to measuring the initial number of DNA DSBs produced after irradiation, the kinetics of the repair of these DNA DSBs are also important. As shown (Figure 2), the repair kinetics for U251 were similar when measured using either the standard immunofluorescent foci assay or the MSD assay. Thus, the MSD assay can be used across a diversity of cell lines to measure the kinetics of γ-H2AX accumulation and dispersal.

**Figure 3:**
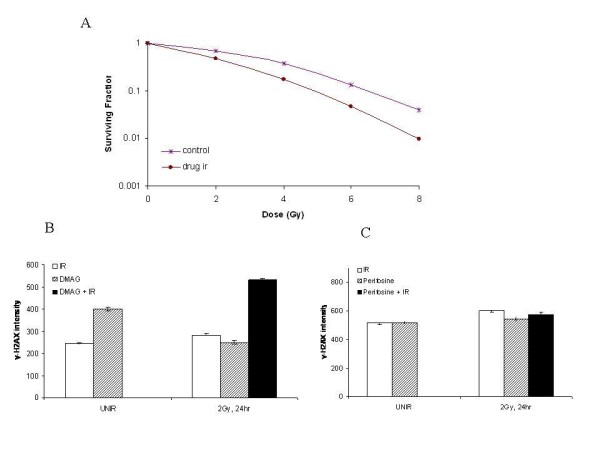
**The effect of drugs on tumor cell radiosensitivity**. (A) U251 Cells were seeded as a single-cell suspension and with a specified number of cells. After allowing cells time to attach (4 h), 17 DMAG or the vehicle control was added (50 nmol/L) and the plates were irradiated 16 h later. Ten to twelve days after seeding, survival curves were generated after normalizing for the cytotoxicity generated by 17 DMAG alone. Data presented are the mean ± SE from at least three independent experiments. (B) Identical experimental conditions as (A) followed by the MSD assay. (C) The non-radiosensitizing effect of perifosine carried out by the MSD assay. U251 cells were treated with perifosine (9 μmol/L) alone and the combination of perifosine and IR.

We have previously published that Hsp90 inhibition enhances tumor cell killing as measured by clonogenic survival, the gold standard for radiation sensitizer development [11]. To demonstrate the potential of the MSD platform to screen drugs as radiation sensitizers, we used this new assay to evaluate the known radiosensitizer 17 DMAG [12]. To determine the effects of the Hsp90 inhibiting drug 17 DMAG on GBM tumor cell radiosensitivity, clonogenic survival analysis was first performed on the U251 cell line. 16 h after 17 DMAG (50 nM) addition, U251 cells were irradiated followed by a change to drug-free media with colony-forming efficiency determined 10 days later. As shown in Figure 3A, this 17 DMAG pretreatment increased U251 radiosensitivity with a dose enhancement factor at a surviving fraction of 0.10 to 1.60, consistent with previous results. In subsequent experiments, U251 cells were exposed to 50 nmol/L of 17 DMAG for 16 hours, irradiated, fed fresh media, and harvested at specific time points. The MSD assay showed retention of γ-H2AX at 24 h post-irradiation in the irradiated cells that were treated with 17 DMAG, which is consistent with non-repair of DNA-DSBs and, thus, a radiosensitizing effect on the GBM tumor cell line matching the clonogenic survival assay (Figure 3B). In addition to the drug 17 DMAG, as a negative control, we investigated a compound, perifosine, known to have no radiomodifying effect. Previous studies using clonogenic survival assays have shown perifosine does not have an effect on repair of DNA-DSBs [13]. Using the MSD assay, we show no increase of γ-H2AX in the combination group compared to the irradiation alone group at 24 h, consistent with the previously published clonogenic assays (Figure 3C).

**Figure 4:**
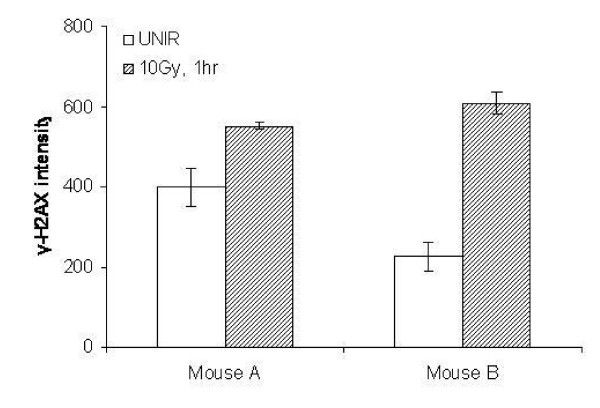
**Evaluation of γ-H2AX *In Vivo***. Tumors were grown in mice injected with U251 cells subcutaneously in the flank. Mice were irradiated, tissue homogenized, protein isolated and the MSD assay was performed.

Finally, the γ-H2AX MSD assay was used on protein isolates from U251 tumors grown subcutaneously in SCID mice, irradiated at 10 Gy and harvested 1 hour post IR. As shown in Figure 4, although there is intra-mouse variability, there is also an increase in γ-H2AX after irradiation demonstrating that the γ-H2AX MSD assay works not only with *in vitro *samples but *in vivo *samples as well. Thus, the γ-H2AX MSD assay can be used as an adjunct to other preclinical assays in evaluating drugs as radiation sensitizers.

Because γ-H2AX expression is an indicator of DSB induction and repair, the development of an analytical method adaptable to a high throughout approach would appear to have a number of applications related to drug development for either radiation sensitizers or for other drugs that kill tumor cells via induction of DNA DSBs. Towards this end, we have demonstrated that the γ-H2AX MSD assay has excellent reproducibility, is quantitative, and applicable to multiple cell types from either *in vitro *or *in vivo *samples. We have also shown that the MSD assay may allow the more rapid development of radiomodifying drugs in a high throughput fashion.

## Competing interests

The authors declare that they have no competing interests.

## Authors' contributions

DA carried out immunofluorescent staining, MSD direct coat assay development and statistical analysis. TS performed the animal studies. WK performed immunofluorescent staining. MS participated in assay development design and execution. PT aided in the overall study design. KC conceived of the study and participated in its design and coordination. All authors read and approved the final manuscript.
